# Application of various machine learning techniques to predict obstructive sleep apnea syndrome severity

**DOI:** 10.1038/s41598-023-33170-7

**Published:** 2023-04-19

**Authors:** Hyewon Han, Junhyoung Oh

**Affiliations:** 1 Department of Computer Engineering, Hongik University, Seoul, 04066 Republic of Korea; 2 Institute for Business Research and Education, Korea University, Seoul, 02841 Republic of Korea

**Keywords:** Sleep disorders, Data mining

## Abstract

As the incidence of obstructive sleep apnea syndrome (OSAS) increases worldwide, the need for a new screening method that can compensate for the shortcomings of the traditional diagnostic method, polysomnography (PSG), is emerging. In this study, data from 4014 patients were used, and both supervised and unsupervised learning methods were used. Clustering was conducted with hierarchical agglomerative clustering, K-means, bisecting K-means algorithm, Gaussian mixture model, and feature engineering was carried out using both medically researched methods and machine learning techniques. For classification, we used gradient boost-based models such as XGBoost, LightGBM, CatBoost, and Random Forest to predict the severity of OSAS. The developed model showed high performance with 88%, 88%, and 91% of classification accuracy for three thresholds for the severity of OSAS: Apnea-Hypopnea Index (AHI) $\ge$ 5, AHI $\ge$ 15, and AHI $\ge$ 30, respectively. The results of this study demonstrate significant evidence of sufficient potential to utilize machine learning in predicting OSAS severity.

## Introduction

Obstructive sleep apnea syndrome(OSAS) is a very common sleep disorder with high prevalence. Globally, nearly 1 billion adults aged 30 to 69 years, are estimated to have mild to severe OSA^1^. OSAS is not only known as a risk factor for hypertension and other various cardiovascular diseases but also to affect the quality of life and cognitive disorders^2–4^. Therefore, active management and treatment are required. Nonetheless, due to the lack of recognition, patients with OSAS symptoms often do not know that they are suffering from OSAS, or even have symptoms of OSAS^5^.

Since the severity of OSAS is estimated using the apnea-hypopnea index(AHI), polysomnography(PSG) is considered as the traditional gold standard for diagnosing OSAS^6,7^. However, PSG requires overnight sleep in a laboratory, a dedicated personnel and system that leads to limited efficiency. In addition, PSG also requires various skin-contacted sensors, which may disturb the subject’s sleep. Other methods are also being attempted to diagnose OSAS, such as home sleep apnea test^8^ and cardiopulmonary monitoring^9,10^, which require at least overnight and also require testing equipment. As the number of suspected OSAS patients increases, the necessity for a simplified new method to countervail the shortcomings of preexisting sleep tests is rising.

As the rapid growth of artificial intelligence affects throughout modern society, applications of artificial intelligence-related technologies have recently emerged in diverse fields. Machine learning, which forms an axis of artificial intelligence, is excellent for recognizing and classifying complex patterns in massive data. This characteristic of machine learning is well-suits to complex, heavy, and enormous healthcare data^11^. Therefore, there is a growing tendency of applying machine learning techniques in medical and healthcare fields^12^. Since variables affecting the morbidity of OSAS and their correlations are complex, machine learning techniques are likely to be appropriate for proposing prediction models.

Since OSAS is a disease with very complex and diverse factors, lots of studies are being conducted to phenotype OSAS. Clustering, a subfield of machine learning and unsupervised learning, is widely used for phenotyping OSAS^13–15^ because it is suitable for multidimensional data without labels. Focusing on this point, this study attempts to obtain better classification performance by proceeding with clustering before classification.

This study aims to present models that can predict the severity of OSAS without performing PSG using assorted machine learning algorithms, in both supervised and unsupervised learning. Since the data is highly dimensional, we attempt to reduce the computation complexity and increase the performance by feature selection and clustering before classification^16^. In this study, experiments are conducted using a variety of methods, from techniques used in machine learning to methods suggested by medical studies. Accuracy is calculated through comparison with AHI measured from actual PSG and through the calculated accuracy, we compare the utility of models according to the severity of OSAS.

## Methods

### Data acquisition and ethics declarations

**Figure 1:**
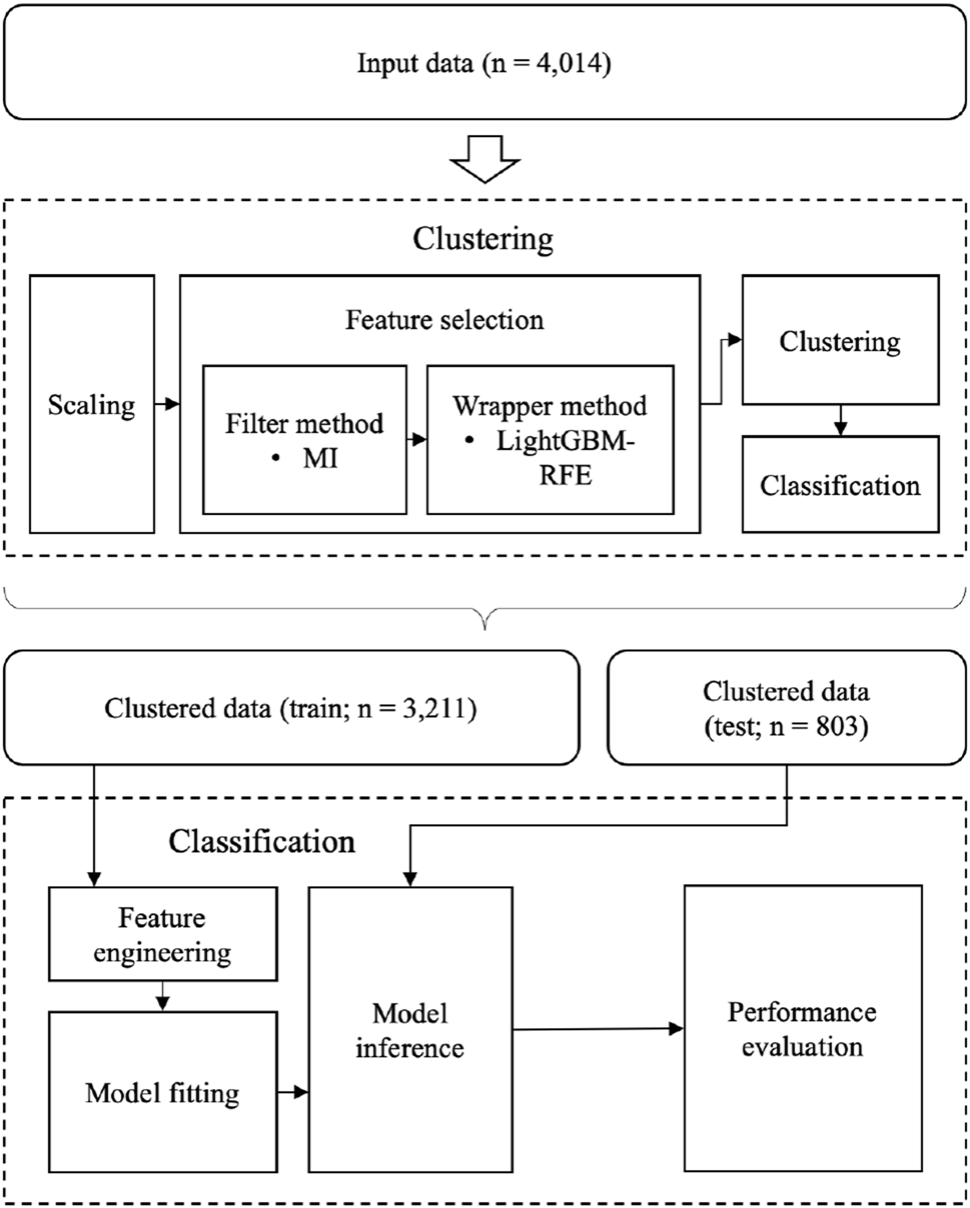
The workflow of the predictive models.

The data used were collected from patients who visited the sleep clinic of Samsung Medical Center between 2014 and 2021. The data include personal information, such as gender, age, height, and weight, as well as physical measurements(abdominal circumference, neck circumference, hip circumference, etc.) and results of self-report questionnaires(Epworth Sleepiness Scale(ESS), Insomnia Severity Index(ISI), etc.) PSG was performed with an Embla N7000 (Medcare-Embla, Reykjavik, Iceland), and the results from the machine’s automated scoring system were used to determine OSAS. AHI was measured as the number of episodes of apnea and hypopnea per hour. PSG features were also collected. The workflow of the predictive models is shown in Fig. 1.

For the software tools, the open-source programming language Python (version 3.9.9; Python Software Foundation, Delaware, USA) was used in all the processes of the study. SciPy^17^ package (version 1.8.1) was mainly used for statistical analysis, and scikit-learn^18^ library (version 1.1.2) was mainly used to develop the predictive models. The study protocol was approved by the institutional review board of Samsung Medical Center (IRB no. 2022-07-003), and the entire process of the study was performed in accordance with the ethical standards of the Declaration of Helsinki. The waiver of informed consent was approved by the institutional review board of Samsung Medical Center since this work is a retrospective study that only involves anonymous patient data.

### Data pre-processing

The processed data consists of 4014 samples and is described by 33 numerical or categorical features. The main characteristics of the dataset are shown in Table 1. The OSAS severity of the dataset was classified into 4 classes corresponding to the severity level defined by the American Academy of Sleep Medicine Task Force^19^. For the classification, 20% of the dataset was used as test data. Each classifier was trained with 5-fold cross-validation with the train dataset. Among input features, numerical features were analyzed for normal distribution using the Kolmogorov-Smirnov’s test. In the case of the normal distribution, Student’s t-test was performed, and in the case of not, the Mann-Whitney U test was conducted. For categorical features, the chi-square test was operated. A *p*-value of less than 0.05 was considered significant.

**Table 1:** The report of statistical analysis. Data are resported as median [interquartile range] or number (percentage). BMI: Body Mass Index, ESS: Epworth Sleepiness Scale, ISI: Insomnia Severity Index, K-BDI-II: Korean-Beck Depression Inventory-II, PSQI: Pittsburgh Sleep Quality Index, SSS: Stanford Sleepiness Scale.

Feature	All (n = 4014)	Training (n = 3211)	Test (n = 803)	*p*-value
Demographic parameters				
Age (years)	53.0 [40.0, 62.0]	53.0 [40.0, 62.0]	54.0 [40.0, 62.0]	0.282
Sex	Male: 2841 (70.78%),	Male: 2289 (71.29%)	Male: 552 (68.74%)	0.956
	Female: 1173 (29.22%)	Female: 922 (28.71%)	Female: 251 (31.26%)	
BMI (kg/$\hbox {m}^2$)	25.2 [23.0, 27.7]	25.1 [23.0, 27.7]	25.3 [22.9, 28.0]	0.387
Height (cm)	168.0 [161.0, 174.0]	168.0 [162.0, 174.0]	168.0 [161.0, 173.0]	0.383
Weight (kg)	71.0 [62.0, 80.0]	71.0 [62.0, 80.0]	71.0 [62.0, 80.0]	0.914
Body measurements				
Abdominal circumference (cm)	90.0 [83.0, 97.0]	90.0 [83.0, 97.0]	90.0 [83.0, 97.0]	0.704
Head circumference (cm)	57.0 [55.0, 58.0]	57.0 [55.0, 58.0]	57.0 [55.0, 58.0]	0.587
Hip circumference (cm)	96.0 [92.0, 100.0]	96.0 [92.0, 100.0]	96.0 [92.0, 100.0]	0.695
Neck circumference (lying position, cm)	38.5 [35.5, 40.5]	38.5 [35.5, 40.5]	38.5 [35.5, 40.5]	0.572
Neck circumference (sitting position, cm)	38.0 [35.0, 40.0]	38.0 [35.0, 40.0]	38.0 [35.0, 40.0]	0.661
Sleep questionnaires				
ESS scores	9.0 [6.0, 13.0]	9.0 [6.0, 13.0]	9.0 [6.0, 13.0]	0.308
ISI scores	11.0 [7.0, 16.0]	11.0 [7.0, 16.0]	12.0 [7.0, 16.0]	0.347
K-BDI-II scores	11.0 [7.0, 17.0]	11.0 [7.0, 17.0]	11.0 [7.0, 17.0]	0.765
PSQI scores	7.0 [5.0, 10.0]	7.0 [5.0, 10.0]	7.0 [5.0, 10.0]	0.636
SSS scores	3.0 [2.0, 3.0]	3.0 [2.0, 3.0]	3.0 [2.0, 3.0]	0.261
Other self-reported parameters				
Hours of sleep	6.0 [5.0, 7.0]	6.0 [5.0, 7.0]	6.0 [5.0, 7.0]	0.755
Consumption of hypnotics	Yes: 484 (12.06%)	Yes: 380 (11.83%)	Yes: 104 (12.95%)	0.973
	No: 3530 (87.94%)	No: 2831 (88.17%)	No: 699 (87.05%)	
PSG parameters				
AHI	20.4 [7.7, 40.4]	20.4 [7.85, 39.8]	20.1 [7.1, 42.45]	0.82
OSAS severity				
Normal	706 (17.59%)	565 (17.6%)	141 (17.56%)	0.261
Mild	897 (22.35%)	717 (21.33%)	180 (22.42%)	
Moderate	971 (24.19%)	777 (24.2%)	194 (24.16%)	
Severe	1440 (35.87%)	1152 (35.88%)	288 (35.87%)	

### Clustering

A combination of mutual information (MI) and recursive feature elimination (RFE)^20^ strategy on LightGBM was applied as feature selection methods for clustering. MI is a metric that indicates the interdependence between two variables, and RFE is a feature selection method that starts with all input features and removes less important features one by one as learning repeats. In the feature selection process, MI was computed to filter less informative variable. The threshold for filtering was set as the mean of the mutual information score. RFE was applied to finally determine the number of features for clustering.

For clustering algorithms, hierarchical agglomerative clustering, K-means, bisecting K-means algorithm, and Gaussian mixture model were used. The algorithms that automatically assign the number of clusters all had a large number of clusters, which did not fit our purpose of conducting clustering. Therefore, clustering algorithms that need to assign the number of clusters manually were used.

Hierarchical clustering is a common clustering algorithm that builds nested clusters by successively merging or splitting them. Agglomerative clustering is a bottom-up approach for hierarchical clustering. Each point starts with an individual cluster and similar clusters are consecutively merged in the clustering process.

K-means is the most popular clustering algorithm^21^ and is known for its simplicity. For finding K clusters, select K points as the initial centroids. Then, assign all points to the nearest centroid and recompute the centroid of each cluster. Repeat these steps until the centroids remain unchanged. Bisecting K-means is a variant of K-means algorithm^22^. Bisecting K-means algorithm uses the basic K-means algorithm to find 2 sub-clusters (bisecting step), and repeats the bisecting step and take the segmentation that produces the clustering with the highest overall similarity.

Gaussian mixture models (GMM) is a probabilistic model which assumes the probability distribution of all subgroups follows the Gaussian distribution^23^.

### Feature engineering

Both methods proposed in medical researches and widely used in machine learning were applied as feature engineering techniques. Weighted ESS and a formula for predicting AHI were used as the medical approach, and body proportion data were also added by processing body measurement data in the dataset.

Weighted ESS is given different weights for each question of ESS. A recent study has shown that weighted ESS is better at predicting the severity of OSAS than general ESS^24^. Since our dataset includes the response of each ESS item, weighted ESS could be applied.

Following is predictive mathematical formula for AHI we used in this work. $\textrm{AHIpred} = \textrm{NC} \times 0.84 + \textrm{EDS} \times 7.78 + \textrm{BMI} \times 0.91 \ - \ [8.2 \times \textrm{gender constant} (1 \textrm{ or } 2) + 37]$^25^. We modified constants using SciPy package to optimize the formula for our dataset. Since the dataset contains two measurements of neck circumference (NC): in sitting and lying positions, the formula was also optimized for those measurements accordingly. In addition, three different criteria were used for determining excessive daytime sleepiness (EDS): the criteria for weighted ESS, the criteria from the American Academy of Sleep Medicine Task Force, and the criteria from the study proposed the predictive formula.

### Predictive models

Gradient boosting-based models and random forest are considered as most effective machine learning models for dealing with large amounts of complex data. These algorithms are proven to be not only accurate but also efficient^26,27^. Therefore, in this work, we used random forest and three different models based on gradient boosting, XGBoost, LightGBM, and CatBoost, to enhance classification performance efficiently.

Random forest is a classifier consisting of a combination of decision trees built on random sub-samples of the dataset^28^. Since the classifier is composed of decorrelated decision trees, it is resistant to noises and the over-fitting problem.

XGBoost is a gradient boosting-based decision tree ensemble designed to be highly efficient and scalable^29^. Since the model automatically operates parallel computation, it is relatively faster than the general gradient boosting framework. XGBoost also lowers the risk of over-fitting by applying different regularization penalties.

LightGBM is a gradient boosting framework designed to be fast and highly efficient^30^. When the data are high-dimensional and large, traditional gradient boosting-based models require scanning all the data instances for each feature to estimate the information gain of all the possible segmentation points, which is excessively time-consuming and inefficient. LightGBM uses Gradient-based One-Side Sampling (GOSS) and Exclusive Feature Bundling (EFB) to deal with this problem. With those techniques, LightGBM reduces the number of samples and the number of features in the dataset.

CatBoost is a gradient boosting on decision trees algorithm that presents an innovative technique to process categorical features, and a variant of gradient boosting which is a permutation-driven alternative^31^. Both methods were created to resist a prediction shift caused by a target leakage, which is present in other implementations of gradient boosting algorithms.

The hyperparameter optimization process is the most cumbersome part of machine learning project. Therefore, diverse optimization techniques are used to simplify the procedure. In this work, we selected Bayesian optimization, which is one of the most commonly used optimization method for hyperparmeter tuning. The hyperparameters to be optimized were selected considering both the characteristics of the dataset and the classifier model. Selected hyperparameters of each model were optimized with a technique based on bayesian optimization using Optuna^32^.

## Results

### Clustering results

**Figure 2:**
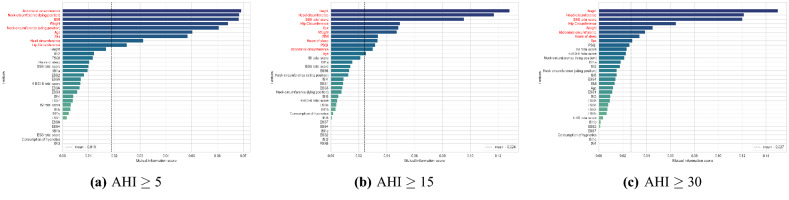
Mutual information (MI) scores for all input features. Each threshold was set as the mean of the mutual information score.

**Figure 3:**
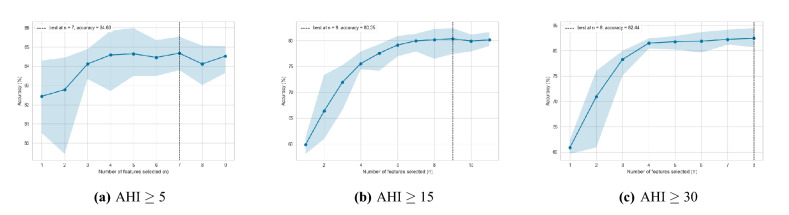
Visualised 5-fold cross-validation results of recursive feature elimination (RFE).

Various feature scaling methods were applied to the numerical features of the dataset and MI-LightGBM-RFE was used for the feature selection. First, MI scores according to AHI cut-off values were computed for all input features to filter out less informative variables. Computed MI scores are shown in Fig. 2. After this process, less important features were eliminated through LightGBM-RFE method. The number of features was determined by the 5-fold cross-validation. Cross-validation result of LightGBM-RFE is shown in Fig. 3. Hip circumference, head circumference, age, neck circumference (sitting position), weight, BMI, abdominal circumference were selected as features for the mild OSAS (AHI $\ge$ 5) clustering. For the moderate OSAS (AHI $\ge$ 15), age, abdominal circumference, PSQI total score, BMI, weight, hip circumference, SSS total score, head circumference, height were selected. For the severe OSAS (AHI $\ge$ 30), sex, hours of sleep, abdominal circumference, weight, hip circumference, SSS total score, head circumference, height were selected.

All of the selected clustering algorithms were applied to datasets of scaled and selected features. The clustering results with the best classification accuracy of the test dataset were selected for the final prediction models. Among the selected clustering algorithms, hierarchical agglomerative clustering recorded the best classification accuracy when the AHI cut-off value is 5. GMM exhibited highest classification accuracy for the moderate OSAS (AHI $\ge$ 15). For the severe OSAS (AHI $\ge$ 30), K-means showed the best performance. The number of clusters was determined using the elbow method based on the silhouette score, and it was determined to be 2 for all AHI cut-off values.

### Classification results by machine learning models and feature engineering methods

**Figure 4:**
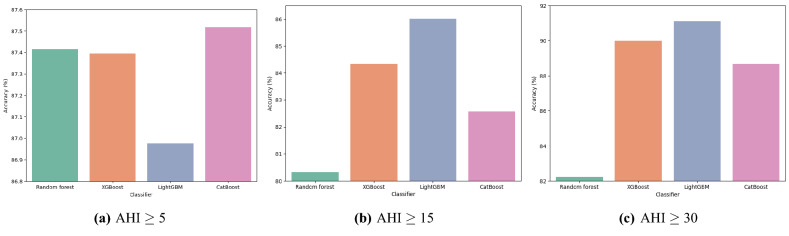
Comparisons of classification accuracy by machine learning classification algorithms.

In the classification accuracy analysis, CatBoost was the best with 87.52% for the mild OSAS. LightGBM recorded the best, achieving 86.01% and 91.11% in the classification of moderate OSAS and severe OSAS, respectively. Figure  4 shows the classification accuracy according to classification algorithms. Overall, LightGBM showed the best performance in all severity classes. On the other hand, Random forest showed the lowest performance in all severity classes showing significant differences from the other machine learning models.

**Figure 5:**
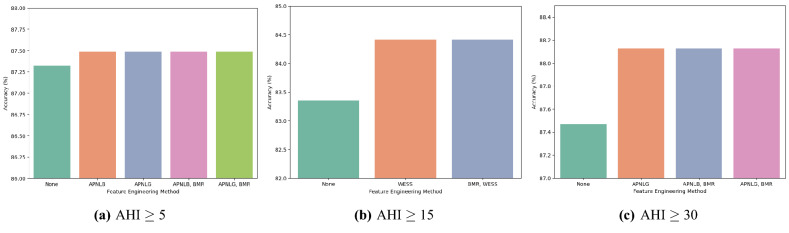
Comparisons of classification accuracy by feature engineering methods. The accuracy of the best performing feature engineering methods and the accuracy of those without the applied feature engineering methods were compared. APNLB: AHI prediction computed using NC in a lying position with EDS criteria from the work of Bouloukaki et al., APNLG: AHI prediction computed using NC in a lying position with general EDS criteria, BMR: Body measurement ratio, WESS: Weighted ESS.

We adopted diverse methods for the dataset in the feature engineering procedure in which all of them were trained and evaluated. For the mild OSAS, applying AHI prediction with neck circumference in a lying position, and applying this method with body measurement ratio showed the best accuracy with 87.48%. For the moderate OSAS, applying weighted ESS, and appying weighted ess with body measurement ratio showed the best accuracy with 84.41%. When predicting the severe OSAS, the best performing feature engineering methods were showed similar with the ones in mild OSAS. The best accuracy was 88.13%. Figure  5 shows the classification accuracy according to feature engineering methods.

### Classification results by approaches building prediction models

**Table 2:** The report of classification metrics of predictive models by approaches. Data are reported as mean (standard deviation) and [score range]. * $p<0.05$ was statistically significant. ** Accuracies of the results were statistically tested and the classification results without clustering were used as the baseline for the statistical test (Mann-Whitney U test).

Predictive model building approach	AHI cut-off value	Accuracy (%)	AUC (%)	f1 (%)	Precision (%)	Recall (%)	*p*-value
Without clustering	5	86.18 (0.10)	84.83 (0.19)	91.98 (0.04)	88.14 (0.49)	96.19 (0.61)	**
		[86.05, 86.30]	[84.55, 85.03]	[91.93, 92.03]	[87.36, 88.64]	[95.47, 97.13]	
	15	81.29 (1.57)	89.93 (1.32)	84.63 (1.18)	83.55 (1.70)	85.74 (0.65)	**
		[78.58, 82.44]	[87.66, 90.85]	[82.59, 85.48]	[80.63, 84.87]	[84.65, 86.31]	
	30	89.13 (3.85)	95.17 (2.56)	83.99 (6.07)	88.32 (4.68)	80.12 (7.19)	**
		[82.57, 92.40]	[90.80, 97.26]	[73.58, 88.93]	[80.58, 93.16]	[67.71, 85.07]	
Clustering only	5	88.14 (0.20)	82.31 (0.67)	93.06 (0.10)	89.11 (0.33)	97.40 (0.31)	${p}<0.05$*
		[87.76, 88.32]	[81.29, 83.40]	[92.90, 93.16]	[88.56, 89.45)	[96.87, 97.71]	
	15	85.75 (1.00)	92.89 (0.19)	89.03 (0.61)	86.43 (1.19)	91.86 (0.26)	${p}<0.05$*
		[84.00, 86.56]	[92.70, 93.24]	[87.96, 89.56]	[84.38, 87.45)	[91.33, 91.99]	
	30	90.74 (0.15)	95.09 (0.16)	78.53 (0.52)	88.42 (0.60)	71.67 (0.89)	0.635
		[90.49, 90.85]	[94.89, 95.22]	[77.54, 78.87]	[87.45, 89.35)	[69.89, 72.27]	
Clustering with feature engineering	5	88.16 (0.25)	82.66 (1.24)	93.11 (0.15)	88.91 (0.32)	97.76 (0.47)	${p}<0.05$*
		[87.76, 88.42]	[80.26, 83.74]	[92.90, 93.28]	[88.56, 89.45)	[96.87, 98.07]	
	15	85.80 (1.51)	92.91 (0.24)	89.08 (0.92)	86.57 (1.67)	91.82 (0.19)	${p}<0.05$*
		[82.78, 86.63]	[92.63, 93.36]	[87.23, 89.60]	[83.24, 87.47)	[91.44, 91.99]	
	30	90.92 (0.20)	95.22 (0.20)	78.76 (0.49)	88.49 (0.79)	72.04 (0.91)	0.278
		[90.58, 91.12]	[94.85, 95.39]	[77.87, 79.20]	[87.12, 89.58)	[70.30, 72.89]	
Clustering with feature engineering and hyperparameter tuning	5	87.82 (0.37)	81.56 (1.55)	92.95 (0.25)	88.56 (0.49)	97.85 (0.87)	${p}<0.05$*
		[87.30, 88.23]	[79.56, 83.81]	[92.65, 93.22]	[88.05, 89.37)	[96.18, 98.55]	
	15	87.84 (1.88)	95.02 (0.44)	90.79 (1.16)	89.27 (2.06)	92.65 (0.00)	${p}<0.05$*
		[85.53, 89.37]	[94.37, 95.76]	[89.37, 91.73]	[86.75, 90.96)	[92.65, 92.65]	
	30	91.06 (1.77)	95.03 (0.83)	75.66 (12.06]	90.87 (3.53)	70.19 (11.63)	0.056
		[87.63, 92.37]	[93.43, 95.73]	[51.62, 82.72]	[86.87, 97.32)	[47.06, 76.77]	

**Figure 6:**
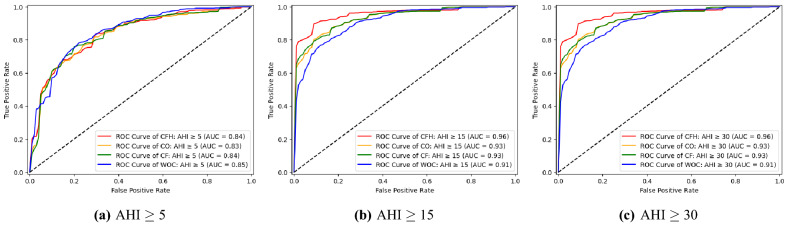
Comparisons of receiver operation characteristic(ROC) curves based on approach to building predictive models. Best records were used for plotting. WOC: Without clustering, CO: Clustering only, CF: Clustering with feature engineering, CFH: Clustering with feature engineering and hyper-parameter tuning.

The prediction results with clustering showed significantly superior performance compared to the prediction results without clustering. The report of classification metrics is presented in Table  2. Statistical significance was tested using the Mann-Whitney U test (significance level 0.05). Using clustering to build a classification model was statistically significant for mild and moderate OSAS classifications compared to without clustering, while it was not for severe OSAS classifications.

In terms of classification accuracy, the approach of clustering with feature engineering and hyperparameter tuning showed the best in moderate and severe OSAS predictions, exhibiting 87.84% and 91.06%, respectively. However, clustering with feature engineering showed the highest accuracy with 88.16% when predicting mild OSAS.

ROC curves according to severity classes of OSAS and approaches to build the predictive models are visualized in Fig.  6. In common with the results of the accuracy analysis, the best AUC value was observed when predicting after clustering with feature engineering and hyperparameter tuning in moderate and severe OSAS predictions. When it comes to predicting mild OSAS, clustering with feature engineering was the best.

## Discussion

In this study, the predictive models for the severity of OSAS were developed by applying various machine learning methodologies. The applicability of the model was tested and analyzed according to the severity. Using MI-LightGBM-RFE, we identified that important features according to each AHI cut-off value for clustering. We also discovered that hierarchical agglomerative clustering, GMM, and K-means clustering are effective for predicting mild OSAS, moderate, and severe OSAS prediction, respectively, based on classification accuracy. Of the three levels of severity, LightGBM performed best for both moderate and severe, except for mild. In particular, it performed well in the moderate OSAS classification, with a fairly large accuracy difference from the other algorithms. While LightGBM is the most functional algorithm overall, CatBoost is the most out-performing algorithm in mild OSAS. Our work demonstrated excellent performances exceeding at least 87% on all three AHI thresholds in classification accuracy.

The gold standard for diagnosing OSAS is PSG. Although, PSG has the disadvantages of being laborious, time-consuming, and expensive. Therefore, many studies have been conducted to develop methods for screening OSAS without performing PSG, and the application of machine learning techniques has also been widely used^33–37^. In recent years, researches on the South Korean population have also been actively conducted. However, there were limitations in that the experiment was conducted on a minority population and focused only on supervised learning^38,39^.

To the best of our knowledge, this work has the best performance among studies predicting OSAS severity from South Korean population using machine learning techniques. Compared to previous studies, this study is significant not only in terms of the research results but also in terms of the research process. In this work, we suggested a new methodology that uses both supervised and unsupervised learning algorithms to predict the severity of OSAS using machine learning techniques. Moreover, our experiment is important in that it has so far targeted the largest South Korean population in the research of predicting OSAS severity using the application of machine learning algorithms.

Despite the appreciable prediction performance, there are several limitations in this study. Since the data were collected from only one sleep clinic, this result is difficult to be estimated for the population of other sleep centers. In addition, a considerable amount of missing values existed in the provided data because this work is a retrospective study.

OSAS is a major worldwide public health concern with an increasing prevalence. Therefore, there is a need for OSAS severity prediction models which can be used in clinical settings. Our work provides the basis for confirming the sufficient potential for utilizing machine learning in OSAS severity prediction, and also suggests outcome prediction models may be useful for screening priorities that assign patients to PSG.

## Conclusion

In this study, we predicted the severity of OSAS with only simple information such as gender and age, body measurement, and questionnaire using diverse machine learning techniques. Compared to the general supervised learning-based machine learning application, the approach of applying machine learning techniques using both supervised and unsupervised learning showed significant performance in OSAS severity prediction. The results of this work demonstrate the superiority of OSAS screening applicability using machine learning methods. Due to the retrospective nature of the study, a considerable amount of data was unavailable for reasons such as missing values, and the data was collected from a single institution, which may introduce bias. Future work could be conducted with data from a larger population at various institutions to improve upon this study. In conclusion, the predictive model presented in this study presents an accurate estimated severity class of OSAS, which provides important evidence that OSAS can be effectively screened without time-consuming and labor-intensive tests.

## Data Availability

The data that support the findings of this study are available from NYX corporation but restrictions apply to the availability of these data, which were used under license for the current study, and so are not publicly available. Data are available from the authors upon reasonable request and with permission of NYX corporation.
